# Polymorphisms in miRNA genes play roles in the initiation and development of cervical cancer

**DOI:** 10.7150/jca.33486

**Published:** 2019-08-20

**Authors:** Zhiling Yan, Ziyun Zhou, Chuanyin Li, Xielang Yang, Longyu Yang, Shuying Dai, Jiehan Zhao, Huijing Ni, Li Shi, Yufeng Yao

**Affiliations:** 1 Institute of Medical Biology, Chinese Academy of Medical Sciences & Peking Union Medical College, Kunming 650118, China; 2 Department of Gynaecologic Oncology, The 3rd Affiliated Hospital of Kunming Medical University, Kunming 650118, China.; 3 School of Basic Medical Science, Kunming Medical University, Kunming 650500, China; 4 Department of Pharmacology, Faculty of Science, University of Alberta, Edmonton, Canada

**Keywords:** microRNA, Polymorphisms, CIN, Cervical cancer, Association, Chinese Han population

## Abstract

MicroRNA deregulation is crucial for cancer development. Studies showed that polymorphisms in miRNA genes could affect miRNA expression, which might be associated with cancer development. In the current study, we investigated the association of seven single nucleotide polymorphisms (SNPs) in seven miRNA genes with the initiation and development of cervical cancer in a Chinese Han population. The SNPs of 358 cervical intraepithelial neoplasia (CIN) patients, 547 cervical cancer patients and 567 healthy individuals were genotyped using TaqMan assays. Moreover, we evaluated the association of the seven SNPs with the different stages of cervical cancer. Our results showed that rs4636297 in miR-126 was associated with susceptibility to CIN and cervical cancer (*P*=0.019 and 0.019, respectively) and that the T allele was associated with a higher risk of CIN (*OR*=1.334, *95% CI*: 1.049-1.698) and cervical cancer (*OR*=1.296, *95% CI*: 1.044-1.609). Similarly, rs11614913 in miR-125a was associated with CIN and cervical cancer (*P*=0.025 and 0.015, respectively), and the T allele might be the protective factor for CIN (*OR*=0.807, *95% CI*: 0.669-0.974) and cervical cancer (*OR*=0.814, *95% CI*: 0.689-0.961). Our results indicated that rs4636297 in miR-126 and rs11614913 in miR-196a2 play an important role only in the initiation of cervical cancer not in the development of CIN to cervical cancer.

## Introduction

Cervical cancer is the second most common malignant tumour among women after breast cancer worldwide^1^. Most cervical cancers (up to 99%) are associated with oncogenic human papillomavirus (HPV)^2^. However, almost all low-risk HPV infections and more than two thirds of high-risk HPV infections are eradicated over a 24-month period^3^, ^4^, and only a small fraction of women infected with HPV will develop cervical cancer^5^. Thus, other factors might also be important during the initiation and development of cervical cancer, such as host genes, reproductive behaviour^6^, sexual activity^7^ and nutritional factors^8^.

MicroRNAs (miRNAs) are a group of short, single-stranded, non-coding RNAs (approximately 18-25 nucleotides in length) that regulate the expression of up to 30% of human genes through targeting the 3′-untranslated region (3′-UTR) or 5′-UTR 9. Therefore, miRNAs are involved in almost every biological process, such as proliferation^10^, apoptosis^11^, migration and invasion^12^. In cervical cancer, abundant miRNAs were found to be restored, and this process was related to cervical cancer development and prognosis^13^, ^14^. The lengths of miRNA genes are usually significantly shorter than those of coding genes; consequently, single nucleotide variations (like single nucleotide polymorphisms, SNPs) in miRNA genes could affect mature progression of miRNAs, resulting in aberrant gene expression^15^, ^16^, which might be the mechanism through which SNPs in miRNA genes are associated with human cancer susceptibility^17^-^22^. In 2016, Wang et al. demonstrated that a polymorphism in miR-155 was associated with cervical cancer risk^23^. Moreover, our previous study showed that rs11134527 in miR-218 and rs531564 in miR-124 were associated with cervical cancer susceptibility in a Chinese Han population^24^.

In the current study, we investigated the distribution of another seven SNPs in miRNA genes (rs543412 in miR-100, rs999885 in miR-106b, rs1143770 in let7-a-2, rs2296616 in miR-107, rs8111742 in miR-125a, rs4636297 in miR-126 and rs11614913 in miR-196a2) in the different steps of cervical cancer (CIN and cervical cancer). Moreover, we analysed the association of these SNPs with the initiation and development of cervical cancer.

## Methods

### Ethical approval and informed consent

All procedures were in accordance with the ethical standards of the responsible committee on human experimentation and with the Helsinki Declaration of 1964, which was revised in 2013. All experimental protocols used in this study were approved by the Institutional Review Boards of the No. 3 Affiliated Hospitals of Kunming Medical University. All participants provided written informed consent.

### Study population

In the current study, 358 patients with CIN and 547 patients with CC were recruited after diagnosis according to “Diagnosis and Treatment Obstetrics and Gynaecology” and FIGO stage (International Federation of Gynaecology and Obstetrics, 2009) at the 3rd Affiliated Hospital of Kunming Medical University from 2012-05 to 2016-08. The inclusion criteria: ① the CIN and cervical cancer patients were diagnosed according to “Diagnosis and Treatment Obstetrics and Gynaecology” and International Federation of Gynaecology and Obstetrics, 2009; ② the patients in case groups were not suffering with any other malignancy, and the control individuals had no history of cancer and other chronic diseases; ③ the patients had not received preoperative neoadjuvant therapies (including chemotherapy and radiotherapy). The exclusion criteria: ① the patients with a prior history of primary cancer other than cervical cancer; ② the patient with malignant tumors except cervical cancer; ③ the patients receiving radiotherapy or chemotherapy, and unclear pathological diagnosis. Over the same period, 567 healthy women from the healthy screening project at the same hospital served as the healthy controls in the current study.

### SNP selection and genotyping

All SNPs selected had minor allele frequencies in the Chinese Han population greater than 5% in the Ensembl database (http://asia.ensembl.org/index.html). SNP-rs999885 is located in the promoter region, while the other SNPs are located in the prI-miRNA sequence. These regions are associated with miRNA gene transcription or miRNA processing and maturation.

Venous blood of the subjects was collected into anticoagulant tubes, and the genomic DNA was extracted from peripheral lymphocytes using a QIAamp Blood Mini Kit (Qiagen, Hilden, Germany). The seven SNPs in the miRNA genes were genotyped using TaqMan Assays. The probes and primers were designed and produced by Thermo Fisher Scientific Company (Waltham, MA, USA), and the TaqMan Master Mix was also from Thermo Fisher Scientific Company. The PCR amplifications were carried out in 384-well reaction plates (MicroAmp™ Optical 384- Well Reaction Plate, Thermo Fisher Scientific Company). The amplification system comprised 2.5μL 2× Master Mix, 0.125 μL 40× primer and probe (FAM and VIC) mix, 1.375 μL ddH_2_O and 1 μL genomic DNA (equivalent ddH_2_O in the negative control). The amplification was conducted in a QuantStudio 6 Flex Fast Real-Time PCR system using the following conditions: 95℃ pre-heat denaturing for 10 min; 92℃ heat denaturing for 10 s and 60 ℃ annealing and extension for 1 min, all repeated for 40 cycles. The data were analysed using QuantStudioTM real-time PCR software (Thermo Fisher Scientific Company). The genotyping results were confirmed through sequencing the SNPs from the subjects with each genotype.

### Statistical analysis

The statistical analyses were performed using SPSS 19.0 software (IBM Corporation, Armonk, New York, USA) and Microsoft Excel (Microsoft Corporation, Redmond, Washington, USA). The representativeness of the subjects in the current study was evaluated using the Hardy-Weinberg equilibrium (HWE). Logistic regression was used to evaluate the effects of the SNPs on the risk of cervical cancer development with age as a covariate, and ORs with 95% confidence intervals (CIs) were calculated. The effects of the SNP genotypes on the risk of cervical cancer were analysed using inheritance model analysis. Three inheritance models, namely, codominant, dominant and recessive, were analysed using SPSS. A *P* value less than 0.05 was considered statistically significant for statistical analysis.

## Results

### Subject characteristics

The characteristics of the individuals enrolled in present study are listed in Table 1. The ages of the CIN, cervical cancer and control groups were not significantly different (*P*>0.05). In the CIN group, 34 patients had low-degree CIN (CIN 1), and 324 had high-degree CIN (CIN 2-3). In the cervical cancer group, there were 447 patients with squamous cell carcinoma (SCC), 87 patients with adenocarcinoma (AC) and 13 patients with other pathological types.

### Association of the seven SNPs with CIN and cervical cancer

The association of these seven SNPs in miRNA genes with CIN and cervical cancer was analysed, and the results calculated using logistic regression are presented in Table 2 and Table 3. The results showed that the T allele of rs4636297 in miR-126 was associated with higher risk of CIN (*P*=0.019; *OR*=1.334, *95% CI*: 1.049-1.698) and cervical cancer (*P*=0.019; *OR*=1.296;* 95% CI*: 1.044-1.349). The T allele of rs11614913 occurred more frequently in the control groups than in the cervical cancer groups (*P*=0.025 and 0.015), and it might be associated with a decreased risk for CIN (*OR*=0.807, *95% CI*: 0.669-0.974) and cervical cancer (*OR*=0.814, *95% CI*: 0.689-0.961). Moreover, the genotypic frequencies for rs4636297 and rs11614913 were significantly different between the CIN and control groups (*P*=0.003 and 0.021, respectively), and between the cervical cancer and control groups (*P*=0.028 and 0.043, respectively).

### Inheritance model analysis of these seven SNPs

Three inheritance models (including codominant, dominant, and recessive) were analysed, and the results are shown in Table 4. The results showed that the TT genotype of rs4636297 was a risk factor for CIN (*OR*=3.611, *95% CI*: 1.624-8.030) and cervical cancer (*OR*=2.343, *95% CI*: 1.056-5.197) compared with C/C-C/T genotype. For rs11614913, the T/C-C/C genotype was associated with a higher risk of CIN (*OR*=1.556,* 95% CI*: 1.126-2.151) and cervical cancer (*OR*=1.343, *95% CI*: 1.018-1.771) compared with the T/T genotype.

## Discussion

Several studies have reported aberrant expression of miRNAs in various human cancers 25, 26. Polymorphisms in miRNA genes could affect miRNA biological processes, resulting in miRNA deregulation that could be associated with cancer development^16^, ^27^. In the current study, we found that rs4636297 in pri-miR-126 and rs11614913 in miR-196a2 were associated with the progression of cervical cancer.

MiR-126 is located in intron 7 of *egfl7* and plays important roles in angiogenesis and inflammation 28-30. In most human cancers, miR-126 functions as a tumour suppressor, and studies have revealed the downregulation of miR-126 in cancerous tissues compared with noncancerous tissues^31^-^34^. In 2008, Wang *et al.* identified the downregulation of miR-126 in cervical cancer^35^. Rs4636297 is located 12 bp downstream of the pre-miR-126 sequence, and this region might be associated with Drosha recognizing and cleaving the pri-miRNA^27^. Thus, this SNP might affect the expression of miR-126, and it could also be associated with human cancers. In the current study, we showed that rs4636297 in the miR-126 gene was associated with CIN and cervical cancer in a Chinese Han population. However, Yang *et al.* did not find an association between this SNP and breast cancer in German women^36^. The discrepancy might be because the two studies selected different populations with different genetic backgrounds. The frequency of the A allele of rs4636297 is 36.4% in the European population, while it is only 18.7% in the East Asian population. The second reason for the discrepancy is that the two studies selected different diseases in which miR-126 might play different roles. However, it will be valuable to carry out functional and associational studies to explore the roles of rs4636297 in human cancers in the future, since the location of this SNP might affect biogenesis.

SNP rs11614913, a polymorphism site in mature miR-196a2, has been widely studied in various human cancers, and the results of such studies indicated that rs11614913 was associated with various human cancers 37-40; however, Zhang et al. found a lack of association between this SNP and gastric cancer^41^. In the current study, our results showed that this SNP was associated with CIN and cervical cancer in the Chinese Han population. Furthermore, the C allele of rs11614913 might be a risk factor for CIN and cervical cancer. Our result was consistent with the Thakur *et al* results^42^. In 2016, Torruella-Loran et al. found that rs11614913 in miR-196a2 has a function in regulating the expression of several genes involved in cancer^43^. SNPs located in the mature sequences of miRNA genes might affect miRNA biogenesis and recognition of target mRNAs^16^. As rs11614913 is located in the mature sequence of miR-196a2, our results indicated that rs11614913 might be associated with cervical cancer in this way.

The roles of miR-107 are different for different cancers. For example miR-107 is a suppressor of breast cancer^44^, ^45^, renal cancer^46^ and glioma^47^; in contrast, this miRNA can promote human gastric cancer^48^, ^49^ and hepatocellular carcinoma^50^. In 2014, Wang et al. found that the TT genotype of rs2296616 was a risk factor for gastric cancer^51^. In addition, they also found that the TT genotype was associated with higher miR-107 expression than the CC genotype^51^. MiR-125a is a suppressor of colon cancer^52^, prostate cancer^53^, ovarian cancer^54^, breast cancer^55^ and prostate cancer^56^. In cervical cancer, miR-125a suppresses tumour growth, invasion and metastasis by targeting STAT3. In 2016, Xu et al. 57 reported that the AA genotype of rs8111742 in miR-125a increased the risk for gastric cancer associated with *H. pylori*, and this same effect was found by Wu et al. 58 in the *H. pylori*-positive group. SNP rs999885, located at the promoter region of the miR-106b-25 cluster, has been reported to be associated with the risk for hepatocellular carcinoma^59^. It was reported that rs1143770 in the let7-a-2 gene is associated with non-small-cell lung cancer^60^ but not gastric cancer^61^. In the current study, we did not find that rs2296616, rs8111742, rs999885 and rs1143770 were associated with cervical cancer. The reasons for these differences between the other studies and the current study could be the various roles of the same miRNAs in different human cancers, and this possibility is supported by the tissue-specific expression of miRNAs^62^. Thus, it is necessary to investigate the function of the SNPs in miRNA genes in specific tissues.

## Conclusion

The current study investigated the association of seven SNPs in miRNA genes (rs543412 in miR-100, rs999885 in miR-106b, rs1143770 in let7-a-2, rs2296616 in miR-107, rs8111742 in miR-125a, rs4636297 in miR-126 and rs11614913 in miR-196a2) with the initiation (control VS CIN) and development of cervical cancer (CIN VS cervical cancer). The results showed that rs4636297 and rs11614913 were associated with the risk of CIN and cervical cancer. However, these two SNPs did not play roles in the progression from CIN to cervical cancer. Therefore, rs4636297 in miR-126, and rs11614913 in miR-196a2, might only be associated with the initiation of cervical cancer, not the development of CIN to cervical cancer.

## Figures and Tables

**Table 1:** The characteristics of the subjects enrolled in the current study

	CIN	Cervical Cancer	Control	*P* value
**N**	358	547	567	
**Ages**	48.22±9.97	48.01±9.53	48.98±7.30	0.125
**CIN1**	34			
**CIN2-3**	324			
**Histological types**				
SCC		447		
AC		87		
Others		13		
**Clinical stages**				
I		307		
II		213		
III		21		
IV		6		
**Parity**				
Yes	350	543	553	
No	8	4	14	
**HPV infection**				
+	354	535		
-	4	12		

**Table 2:** Allelic distribution of the SNPs in control, CIN and cervical cancer groups

SNPs	Alleles (*n*,%)		Control VS CIN		Control VS Cervical cancer		CIN VS Cervical cancer	
			*P* value	*OR[95%CI]*	*P* value	*OR[95%CI]*	*P* value	*OR[95%CI]*
**rs543412**	C	T						
Control	677(59.7%)	457(40.3%)	0.261	0.895[0.738-1.086]	0.765	0.974[0.822-1.155]	0.422	1.083[0.892-1.314]
CIN	446(62.3%)	270(37.7%)						
Cervical cancer	661(60.4%)	433(39.6%)						
**rs999885**	A	G						
Control	895(81.3%)	239(18.7%)	0.923	0.989[0.785-1.246]	0.164	0.862[0.699-1.062]	0.278	0.877[0.692-1.111]
CIN	568(79.3%)	148(20.7%)						
Cervical cancer	890(81.4%)	204(18.6%)						
**rs1143770**	C	T						
Control	528(46.6%)	606(53.4%)	0.804	0.976[0.809-1.179]	0.834	0.982[0.831-1.161]	0.921	1.010[0.836-1.220]
CIN	329(46.0%)	387(54.0%)						
Cervical cancer	505(46.2%)	589(53.8%)						
**rs2296616**	T	C						
Control	1052(92.8%)	82(7.2%)	0.567	1.109[0.779-1.578]	0.849	0.969[0.704-1.334]	0.668	1.080[0.759-1.537]
CIN	659(92.0%)	57(8.0%)						
Cervical cancer	1013(92.6%)	81(7.4%)						
**rs8111742**	A	G						
Control	341(30.1%)	793(69.9%)	0.167	1.153[0.942-1.410]	0.191	1.127[0.942-1.349]	0.785	0.973[0.796-1.188]
CIN	238(32.4%)	478(67.6%)						
Cervical cancer	357(32.6%)	737(67.4%)						
**rs4636297**	C	T						
Control	948(83.6%)	186(16.4%)	0.019	1.334[1.049-1.698]	0.019	1.296[1.044-1.609]	0.785	0.973[0.770-1.229]
CIN	569(79.5%	147(20.5%)						
Cervical cancer	874(79.9%)	220(20.1%)						
**rs11614913**	C	T						
Control	546(48.1%)	588(51.9%)	0.025	0.807[0.669-0.974]	0.015	0.814[0.689-0.961]	0.938	1.008[0.834-1.217]
CIN	383(53.5%)	333(46.5%)						
Cervical cancer	583(53.3%)	511(46.7%)						

**Table 3:** Genotypic distribution of the seven SNPs in Control, CIN and Cervical cancer groups

SNPs	Genotypes (*n*, %)			*P* Value		
				Control VS CIN	Control VS Cervical cancer	CIN VS Cervical cancer
**rs543412**	C/C	C/T	T/T			
Control	199(35.1%)	279(49.2%)	89(15.7%)	0.253	0.739	0.577
CIN	144(40.2%)	158(44.1%)	56(15.6%)			
Cervical cancer	203(37.1%)	255(46.6%)	89(16.3%)			
**rs999885**	A/A	A/G	G/G			
Control	350(61.7%)	195(34.4%)	22(3.9%)	0.159	0.112	0.542
CIN	231(64.5%)	106(29.6%)	21(5.9%)			
Cervical cancer	367(67.1%)	156(28.5%)	24(4.4%)			
**rs1143770**	C/C	C/T	T/T			
Control	118(20.8%)	292(51.5%)	157(27.7%)	0.741	0.562	0.979
CIN	77(21.5%)	175(48.9%)	106(29.6%)			
Cervical cancer	121(22.1%)	263(48.1%)	163(29.8%)			
**rs2296616**	T/T	T/C	C/C			
Control	490(86.4%)	72(12.7%)	5(0.9%)	0.291	0.447	0.852
CIN	302(84.4%)	55(15.4%)	1(0.3%)			
Cervical cancer	468(85.5%)	77(14.1%)	2(0.4%)			
**rs8111742**	A/A	A/G	G/G			
Control	48(8.5%)	245(43.2%)	274(48.3%)	0.073	0.419	0.423
CIN	47(13.1%)	144(40.2%)	167(46.6%)			
Cervical cancer	60(11.0%)	237(43.3%)	250(45.7%)			
**rs4636297**	C/C	C/T	T/T			
Control	390(68.8%)	168(29.6%)	9(5.6%)	0.003	0.028	0.289
CIN	231(64.5%)	107(29.9%)	20(5.6%)			
Cervical cancer	347(63.4%)	180(32.9%)	20(3.7%)			
**rs11614913**	T/T	T/C	C/C			
Control	153(27.0%)	282(49.7%)	132(23.3%)	0.021	0.043	0.442
CIN	68(19.0%)	197(55.0%)	93(26.0%)			
Cervical cancer	117(21.4%)	277(50.6%)	153(28.0%)			

**Table 4:** The inheritance model analysis of the seven SNPs in miRNA genes among Control, CIN and Cervical cancer groups

SNPs	Models	Genotypes	Control (*n*,%)	CIN (*n*,%)	Cervical cancer (*n*,%)	CIN VS Control		Cervical cancer VS Control		Cervical cancer VS CIN	
						*OR[95%CI]*	*P* value	*OR[95%CI]*	*P* value	*OR[95%CI]*	*P* value
**rs543412**	Codominant	C/C	199(35.1%)	144(40.2%)	203(37.1%)	1	0.259	1	0.724	1	0.641
		C/T	279(49.2%)	158(44.1%)	255(46.6%)	0.784[0.587-1.048]		0.904[0.697-1.172]		1.145[0.855-1.532]	
		T/T	89(15.7%)	56(15.6%)	89(16.3%)	0.863[0.580-1.285]		0.985[0.692-1.404]		1.127[0.758-1.676]	
	Dominant	C/C	199(35.1%)	144(40.2%)	203(37.1%)	1	0.116	1	0.527	1	0.347
		C/T-T-T	368(64.9%)	214(59.8%)	344(62.9%)	0.804[0612-1.056]		0.924[0.723-1.181]		1.140[0.867-1.499]	
	Recessive	C/C-C-T	478(84.3%)	302(84.4%)	458(83.7%)	1	0.947	1	0.794	1	0.800
		T/T	89(15.7%)	56(15.6%)	89(16.3%)	0988[0.686-1.422]		1.044[0.757-1.439]		1.048[0.728-1.509]	
**rs999885**	Codominant	A/A	350(61.7%)	231(64.5%)	367(67.1%)	1	0.170	1	0.101	1	0.552
		A/G	195(34.4%)	106(29.6%)	156(28.5%)	0.826[0.619-1.104]		0.760[0.588-0.983]		0.927[0.689-1.248]	
		G/G	22(3.9%)	21(5.9%)	24(4.4%)	1.442[0.775-2.683]		1.056[0.581-1.921]		0.725[0.394-1.334]	
	Dominant	A/A	350(61.7%)	231(64.5%)	367(67.1%)	1	0.402	1	0.061	1	0.435
		A/G-G/G	217(38.3%)	127(35.5%)	180(32.9%)	0.889[0.675-1.170]		0.790[0.618-1.011]		0.894[0.675-1.184]	
	Recessive	A/A-A/G	545(96.1%)	337(94.1%)	523(95.6%)	1	0.170	1	0.855	1	0.332
		G/G	22(3.9%)	21(5.9%)	24(4.4%)	1.537[0.832-2.839]		1.155[0.639-2.088]		0.742[0.406-1.358]	
**rs1143770**	Codominant	C/C	118(20.8%)	77(21.5%)	121(22.1%)	1	0.741	1	0.504	1	0.964
		C/T	292(51.5%)	175(48.9%)	263(48.1%)	0.925[0.656-1.304]		0.877[0.647-1.189]		0.956[0.678-1.348]	
		T/T	157(27.7%)	106(29.6%)	163(29.8%)	1.041[0.713-1.520]		1.016[0.726-1.421]		0.981[0.673-1.429]	
	Dominant	C/C	118(20.8%)	77(21.5%)	121(22.1%)	1	0.832	1	0.596	1	0.832
		C/T-T/T	449(79.2%)	281(78.5%)	426(77.9%)	0.966[0.0.698-1.335]		0.925[0.695-1.233]		0.966[0.698-1.335]	
	Recessive	C/C-C/T	410(72.3%)	252(70.4%)	384(71.2%)	1	0.525	1	0.419	1	0.419
		T/T	157(27.7%)	106(29.6%)	163(29.8%)	1.099[0.821-1.473]		1.113[0.858-1.444]		1.310[0.858-1.444]	
**rs2296616**	Codominant	T/T	490(86.4%)	302(84.4%)	468(85.5%)	1	0.305	1	0.467	1	0.848
		T/C	72(12.7%)	55(15.4%)	77(14.1%)	1.245[0.852-1.820]		1.129[0.799-1.596]		0.903[0.621-1.315]	
		C/C	5(0.9%)	1(0.3%)	2(0.4%)	0.327[0.038-2.818]		1.016[0.726-1.421]		1.290[0.116-14.292]	
	Dominant	T/T	490(86.4%)	302(84.4%)	468(85.5%)	1	0.371	1	0.640	1	0.620
		T/C-C/C	77(13.6%)	56(15.6%)	79(14.5%)	1.186[0.816-1.723]		1.084[0.772 -1.522]		0.910[0.628-1.320]	
	Recessive	T/T-T/C	562(99.1%)	357(99.7%)	545(99.6%)	1	0.296	1	0.306	1	0.826
		C/C	5(0.9%)	1(0.3%)	4(0.4%)	0.317[0.037-2.730]		0.424[0.082-2.195]		1.310[0.118-14.498]	
**rs8111742**	Codominant	A/A	48(8.5%)	47(13.1%)	60(11.0%)	1	0.089	1	0.309	1	0.501
		A/G	245(43.2%)	144(40.2%)	237(43.3%)	0.610[0.388-0.959]		0.762[0.501-1.161]		1.289[0.835-1.990]	
		G/G	274(48.3%)	167(46.6%)	250(45.7%)	0.632[0.404-0.988]		0.721[0.475-1.095]		1.173[0.763-1.801]	
	Dominant	A/A	48(8.5%)	44(12.3%)	60(11.0%)	1	0.058	1	0.142	1	0.326
		A/G-G/G	519(91.5%)	314(87.7%)	487(89.0%)	0.661[0.435-1.023]		0.741[0.496-1.105]		1.226[0.816-1.843]	
	Recessive	A/A-A/G	293(51.6%)	188(53.4%)	297(54.3%)	1	0.794	1	0.385	1	0.781
		G/G	274(48.3%)	170(46.6%)	250(45.7%)	0.937[0.719-1.222]		0.901[0.711-1.140]		0.963[0.737-1.258]	
**rs4636297**	Codominant	C/C	390(68.8%)	231(64.5%)	347(63.4%)	1	0.006	1	0.036	1	0.288
		C/T	168(29.6%)	107(29.9%)	180(32.9%)	1.085[0.810-1.454]		1.216[0.941-1.571]		1.122[0.838-1.502]	
		T/T	9(5.6%)	20(5.6%)	20(3.7%)	3.703[1.657-8.275]		2.494[1.120-5.557]		0.665[0.350-1.263]	
	Dominant	C/C	390(68.8%)	231(64.5%)	347(63.4%)	1	0.163	1		1	0.732
		C/T-T/T	177(31.2%)	127(35.5%)	200(36.6%)	1.221[0.922-1.617]		1.282[0.999-1.644]		1.050[0.795-1.386]	
	Recessive	C/C-C/T	558(98.4%)	338(94.4%)	527(96.3%)	1	0.002	1	0.036	1	0.168
		T/T	39(1.6%)	20(5.6%)	20(3.7%)	3.611[1.624-8.030]		2.343[1.056-5.197]		0.640[0.339-1.208]	
**rs11614913**	Codominant	T/T	153(27.0%)	68(19.0%)	117(21.4%)	1	0.028	1	0.043	1	0.417
		T/C	282(49.7%)	197(55.0%)	277(50.6%)	1.547[1.101-2.174]		1.268[0.946-1.700]		0.816[0.574-1.158]	
		C/C	132(23.3%)	93(26.0%)	153(28.0%)	1.575[1.066-2.327]		1.503[1.074-2.102]		0.955[0.644-1.418]	
	Dominant	T/T	153(27.0%)	68(19.0%)	117(21.4%)	1	0.007	1	0.037	1	0.377
		T/C-C/C	414(73.0%)	290(81.0%)	430(78.6%)	1.556[1.126-2.151]		1.343[1.018-1.771]		0.860[0.616-1.202]	
	Recessive	T/T-T/C	435(76.7%)	265(74.0%)	394(72.0%)	1	0.337	1	0.074	1	0.508
		C/C	132(23.3%)	93(26.0%)	153(28.0%)	1.162[0.855-1.579]		1.280[0.977-1.677]		1.107[0.819-1.498]	
